# Neural Stem Cells in *Drosophila*: Molecular Genetic Mechanisms Underlying Normal Neural Proliferation and Abnormal Brain Tumor Formation

**DOI:** 10.1155/2012/486169

**Published:** 2012-06-07

**Authors:** Nidhi Saini, Heinrich Reichert

**Affiliations:** Biozentrum, University of Basel, 4056 Basel, Switzerland

## Abstract

Neural stem cells in *Drosophila* are currently one of the best model systems for understanding stem cell biology during normal development and during abnormal development of stem cell-derived brain tumors. In *Drosophila* brain development, the proliferative activity of neural stem cells called neuroblasts gives rise to both the optic lobe and the central brain ganglia, and asymmetric cell divisions are key features of this proliferation. The molecular mechanisms that underlie the asymmetric cell divisions by which these neuroblasts self-renew and generate lineages of differentiating progeny have been studied extensively and involve two major protein complexes, the apical complex which maintains polarity and controls spindle orientation and the basal complex which is comprised of cell fate determinants and their adaptors that are segregated into the differentiating daughter cells during mitosis. Recent molecular genetic work has established *Drosophila* neuroblasts as a model for neural stem cell-derived tumors in which perturbation of key molecular mechanisms that control neuroblast proliferation and the asymmetric segregation of cell fate determinants lead to brain tumor formation. Identification of novel candidate genes that control neuroblast self-renewal and differentiation as well as functional analysis of these genes in normal and tumorigenic conditions in a tissue-specific manner is now possible through genome-wide transgenic RNAi screens. These cellular and molecular findings in *Drosophila* are likely to provide valuable genetic links for analyzing mammalian neural stem cells and tumor biology.

## 1. Introduction

Stem cells play a central role in the process of growth and development in multicellular organisms in which they ensure the generation of a large and diverse set of cell types as well as provide for the maintenance of tissue homeostasis [1–3]. In recent years stem cells in the genetic model system *Drosophila* have become an excellent model for studying the cellular and molecular mechanisms that underlie stem cell function. Specifically, the neural stem cells in *Drosophila*, called neuroblasts for historical reasons, are currently one of the best and most extensively used model systems for understanding stem cell biology during normal development [4, 5]. Moreover, *Drosophila* neural stem cells have also become useful for understanding the cellular and molecular basis of stem cell-derived brain tumors that arise due to loss of control of the stem cell divisions [6, 7]. In this review, we focus on the cellular mechanisms of neural stem cell proliferation in the central brain and optic lobes of *Drosophila* under normal conditions, present the current state of insight into the molecular elements that control the proliferative action of these neural stem cells during brain development, and discuss the alterations in the mechanisms of neural stem cell control that lead to overproliferation and brain tumor formation.

## 2. Neural Stem Cells in *Drosophila*: Neuroblasts of the Central Brain and Optic Lobe

**Figure 1:**
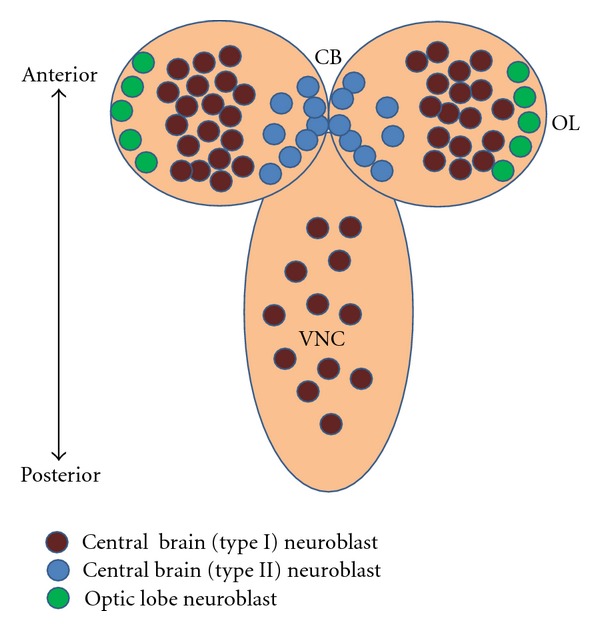
Schematic representation of development of the nervous system in the third instar *Drosophila* larval brain. During postembryonic neuroblast development, the brain of *Drosophila* can be divided into the paired optic lobes (OL) at the lateral surface of the two hemispheres, the central brain (CB), located medially to the OL, and the ventral nerve cord (VNC). The type I neuroblasts are the most abundant in the CB and VNC. The type II neuroblasts are located on the dorsomedial surface of the hemispheres.

**Figure 2:**
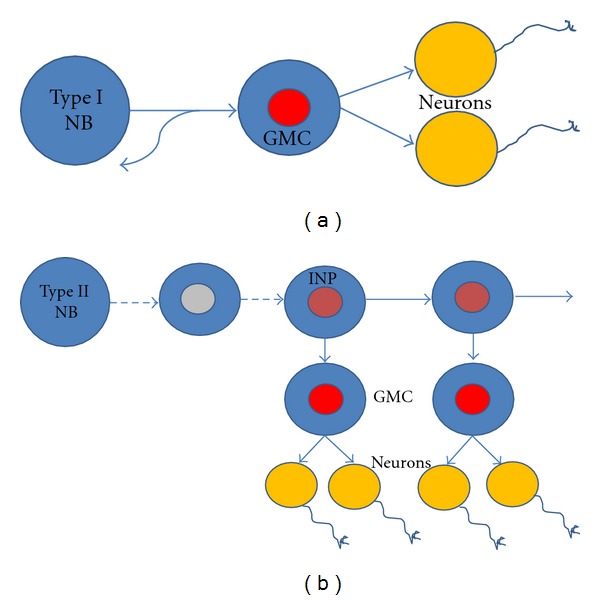
Neural stem cells/neuroblast (NB) undergo two types of self-renewing cell divisions: symmetric (proliferating) and/or asymmetric (differentiating). (a) Type I NB self-renew, and also generates a ganglion mother cell (GMC) which divides only once to generate two postmitotic daughter cells that differentiate into neurons or glial cells [2, 16–18]. (b) Type II NB initiates expression of the proneural gene *asense* and becomes an intermediate neural precursor (INP), which undergoes self-renewing asymmetric divisions, with each division resulting in one INP and one GMC [22–26]. Type II NB generates much larger lineages compared to type I NB.

The brain of *Drosophila* can be divided into the paired optic lobes and the central brain, and the neurons in both of these structures derive from neuroblasts. Of these two sets of neuroblasts, the neuroblasts that give rise to the central brain have been studied in much greater detail (Figure 1). There are two kinds of central brain neuroblasts, type I and type II. The more abundant type I neuroblasts delaminate from the ventral cephalic neuroectoderm during embryogenesis and undergo up to 20 rounds of proliferative activity to generate the restricted number of neurons that make up the larval brain. Subsequently these neuroblasts enter quiescence by embryonic stage 16 and later during larval development in the second instar larval stage, and they re-enter the cell cycle to generate the vast majority neurons of the adult brain [8–15]. The proliferative activity of most central brain neuroblasts during embryonic and postembryonic stages is comparable and relies on asymmetric cell divisions by which the neuroblasts self-renew and also generate a smaller daughter called ganglion mother cell (GMC) which undergoes a single cell division to generate two postmitotic daughter cells that differentiate into neurons or glial cells [2, 16–18] (Figure 2(a)). Other specialized kinds of type I NB are found in the mushroom bodies and the optic lobes [19–21].

In addition to the majority of these so-called type I neuroblasts a smaller set of type II neuroblasts is located in the dorsoposterior and medioposterior region of each of the two central brain hemispheres (8 per hemisphere); these neuroblasts manifest a somewhat different proliferative activity that shows an interesting amplification of neural proliferation. Unlike the type I neuroblasts, in type II neuroblast proliferation the smaller daughter cell initiates expression of the proneural gene *asense* and becomes an intermediate neural precursor (INP), which undergoes a limited number of repeated self-renewing asymmetric divisions, with each division resulting in one INP and one GMC [22–26] (Figure 2(b)). Due to the amplification of proliferation through INPs, the type II neuroblasts can produce lineages of neurons which are markedly larger in size than those of type I neuroblasts.

**Figure 3:**
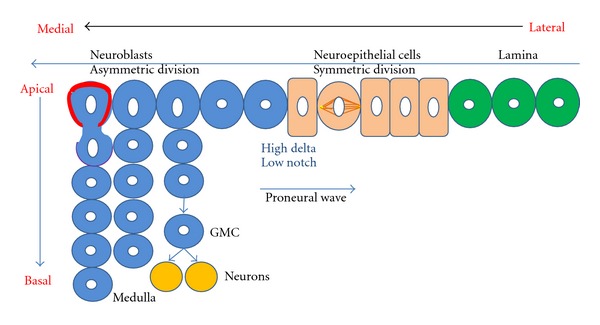
Schematic representation of neurogenesis in optic lobe development. During larval development transition from neuroepithelial (NE, orange) to neuroblast (NB, blue) takes place. NE cells undergo symmetric proliferation with a horizontal spindle orientation to expand the pool of precursor cells and give rise to asymmetrically dividing NB (green). This is in response to the proneural wave of *lethal of scute*. The median NB divides asymmetrically with a vertical spindle orientation, owing to the clear subcellular localization of the apical (polarity proteins, red boundary) and basal (cell-fate determinants and their adaptor proteins, purple boundary) complex, to give rise to the Ganglion mother cells (GMCs) and further, post mitotic neuronal daughter cells. Most central brain neuroblasts during embryonic and postembryonic stages undergo asymmetric cell divisions [21, 27, 28].

The neuroblasts of the optic lobes also derive from neuroectodermal cells; however, the development of the optic lobe neuroectoderm and the manner in which the optic lobe neuroblasts differentiate from this neuroectoderm are different from the situation in the central brain. The optic lobes derive from an embryonic optic placode, which during larval stages form two proliferation centers adjacent to the central brain, the inner optic anlagen and the outer optic anlagen. In the inner optic anlagen, neuroepithelial cells initially divide symmetrically to expand the pool of potential precursor cells and later on transform into optic lobe neuroblasts in an ordered and highly localized manner in response to a wave of proneural gene expression that traverses the neuroepithelium [19, 35–37]. Subsequent to their formation, the optic lobe neuroblasts switch to a neurogenic mode and proliferate by undergoing a limited number of asymmetrical cell divisions which generate neuronal progeny in a manner that is similar, but not identical, to that of the asymmetrically dividing neuroblasts in the central brain [21, 27, 28, 38] (Figure 3).

## 3. Molecular Mechanisms for Neural Proliferation in Central Brain and Optic Neuroblasts

The molecular mechanisms that underlie the asymmetrical cell divisions by which neural stem cells self-renew and generate lineages of differentiating progeny have been studied extensively in the neuroblasts of the central brain [2, 39]. From a temporal point of view, each asymmetric cell division can be divided into three successive steps, namely, establishment of a polarity axis during interphase, followed by appropriate spindle orientation during the onset of mitosis and finally by asymmetric localization of cell fate determinants in the neuroblast and their inheritance by only one of the two daughter cells at the end of mitosis [40, 41]. From a molecular point of view these successive steps involve two major protein complexes: the apical complex and the basal complex.

**Figure 4:**
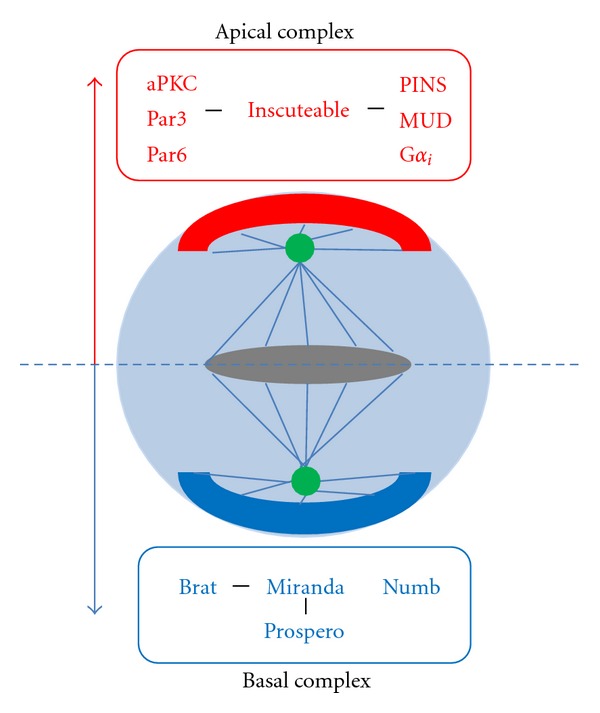
Asymmetric cell division in *Drosophila* neuroblasts. Apical (red) and basal (blue) proteins are asymmetrically segregated at cortical ends of the neuroblast at the time of mitosis. Members of the apical complex are involved in the asymmetric partitioning of basal determinants, in establishing cell polarity and in the correct orientation of the mitotic spindle. The apical complex consisting of aPKC, Par3, and Par6 is linked to the G*α*i-PINS-MUD complex via Inscuteable. The basal complex consists of the cell-fate determinants, Miranda, Prospero, Brat, and Numb.

Members of the apical complex include the PDZ domain-containing proteins PAR3 and PAR6 and the protein kinase atypical PKC (aPKC) [42–48] which accumulate at the apical cell cortex prior to mitosis and are also involved in the asymmetric partitioning of basal determinants [2, 49]. Other proteins constituting this complex are the adaptor protein, Inscuteable [50, 51] which links PAR3-PAR6-aPKC to a further protein complex containing the heterotrimeric G protein *α*_*i*_-subunit, G*α*_*i*_ [52–55], and the adaptor protein Partner of Inscuteable, PINS [56–58]. The PINS protein interacts with the microtubule-associated dynein-binding protein, MUD providing for a cortical attachment site for astral microtubules which maintains the apical-basal orientation of the mitotic spindle [59–61]. *Drosophila* neuroblasts have asymmetrically shaped mitotic spindles, where the apical microtubule asters are larger than their basal counterparts and this contributes to asymmetric cell division since it results in two different sized daughter cells [51, 62, 63]. Interestingly, the site of cytokinesis has recently been shown to be determined by another cortical pathway which is mediated by the apical PINS-G*α*_*i*_-MUD complex. Here the cleavage furrow proteins and the myosin segregates into the basal part of the cell even before the mitotic spindle assumes asymmetry. Moreover, in mutants with abnormal spindle orientation but normal cortical polarity, or in flies where spindle formation is blocked, the cortical asymmetry and the resulting cleavage furrow still establishes itself normally [49, 64] (Figure 4).

Members of the basal complex include the cell-fate determinants Numb, Prospero, and Brat which are asymmetrically segregated into the GMC during neuroblast division [4, 5, 7, 65–67]. During mitosis, these cell-fate determinants are transiently concentrated in a basal cortical crescent in the neuroblast and are subsequently segregated asymmetrically into the GMCs. The endocytic protein Numb is a tissue-specific inhibitor of Notch-Delta signaling and was the first asymmetrically segregating cell fate determinant characterized in *Drosophila* [68–73]. The translational inhibitor Brat (brain tumor) as well as (in type I neuroblasts) the homeodomain transcription factor Prospero is also asymmetrically segregated into the GMC aided by the adaptor protein Miranda [29–32, 74–80] (Figure 4). In the GMC, Prospero translocates to the nucleus where it represses the cell-cycle genes and induces neuronal differentiation genes. Brat is thought to act both as a translational repressor and an inhibitor of cell growth as well as a regulator of the transcription factor Myc and micro-RNAs; however, the precise mechanisms by which Brat regulates cell fate is not known [80–82]. In contrast to type I neuroblasts, the Asense-negative type II neuroblasts do not express Prospero, hence, Prospero is not segregated to the INP daughter cell during type II neuroblast division and this may contribute to the continued proliferative activity of INPs in these lineages. This restricted proliferative potential of INPs during limited rounds of asymmetric divisions is maintained by the transcription factor Earmuff [26, 75].

The molecular mechanisms that control the limited number of asymmetric proliferative divisions of the neuroblasts in the optic lobe are thought to be similar to those that operate in the INPs of type II neuroblast lineages in the central brain, however, this has not yet been studied in more detail. In contrast, a considerable amount of information is available on the molecular control of the neuroectoderm to neuroblast transformation occurring in the developing optic lobe. Initially and prior to neuroblast formation, the neuroectodermal cells are maintained in their expansive symmetrical division mode by Notch signaling, which also prevents their transformation to neuroblasts [83, 84]. However, at the spatially dynamic transition zone between epithelial neuroectodermal cells and neuroblasts, Notch activity is reduced and high levels of Delta are observed [85–87]. The transition between neuroepithelial cells and neuroblasts takes place in response to a proneural wave of *lethal of scute* (*l'sc*) expression which sweeps across the neuroepithelium and leaves the asymmetrically dividing neuroblasts behind it [38, 88] (Figure 3). JAK/STAT and EGFR pathways are involved in the control of this wave's progression [85]. Moreover, the differentiation of neuroepithelial cells into neuroblasts at this zone has been shown to involve the Salvador-Warts-Hippo (SWH) signaling pathway [86]. It is noteworthy that this transition from symmetrically dividing neuroepithelial cells to asymmetrically dividing neuroblasts is similar to the transition from self-renewing to neurogenic neural stem cells in mammalian cortical development.

## 4. Abnormal Neuroblast Proliferation and Brain-Tumor Formation

Classical genetic screens have identified a number of genes such as *brat*, *l(2)gl*, *dlg*, *lethal (2) giant discs,* and *lethal (3) malignant brain tumor* as potent tumor suppressor genes. Flies mutated for any of these tumor suppressor genes develop a tumor-like overproliferation in tissues such as the brain or the imaginal discs [39, 89–93]. Building on these classical genetic studies, and based on the cellular and molecular analysis of the proliferation of neuroblasts under normal conditions, recent molecular genetic work has now established *Drosophila* neuroblasts as an excellent model system for understanding the mechanisms that underlie neural stem cell-derived tumors [4, 5]. Interestingly, these recent investigations have shown that both the molecular mechanisms that control asymmetric cell divisions of neuroblasts in the central brain and those that control the neuroectodermal expansion/transition in the optic lobes are prone to dysregulation which can lead to brain tumor formation.

**Figure 5:**
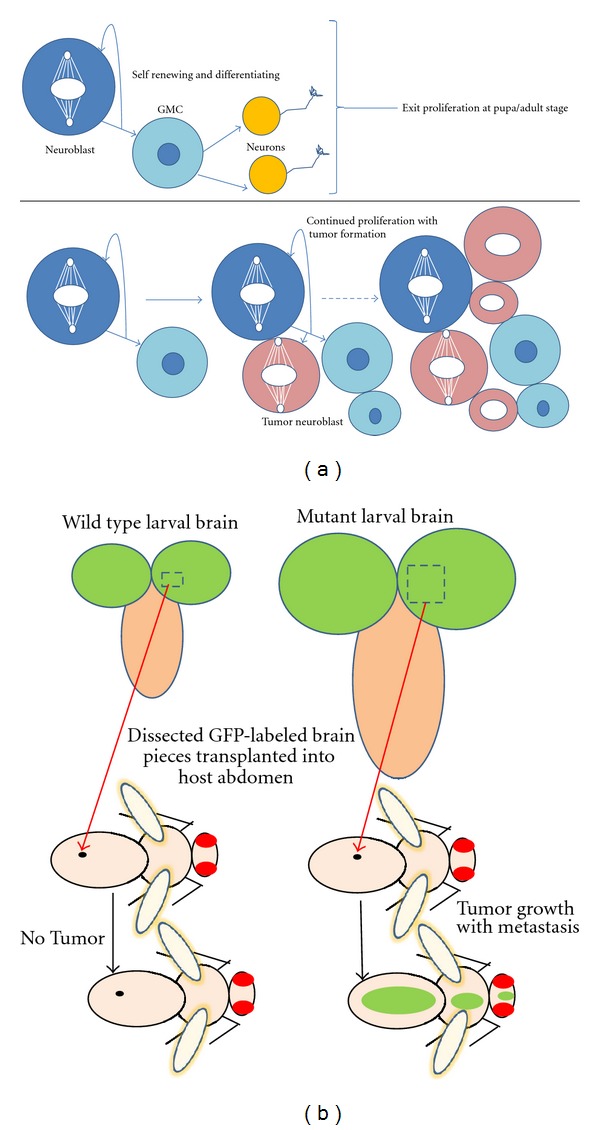
Abnormal neuroblast proliferation and brain tumor formation. (a) (Top), wild-type *Drosophilae* have “normal neuroblasts” which undergo a regulated self-renewal and differentiation process to generate neurons or glial cells. This proliferation exits at pupal stage. (Bottom), Dysregulated asymmetric cell division in central brain neuroblasts of larval brains with knockdown or knockout of cell-fate determinants results in brain tumor formation [24, 29–32]. The disturbed balance between self-renewal and differentiation results in the generation of self-renewing “tumor neuroblasts” and indefinite proliferation. (b) (Left) wild-type larval brain compared to (right) cell fate determinant (*brat*/*prospero*/*numb*) mutant, overproliferated brain. Transplantation of dissected GFP-labeled neuroblasts from the latter results in tumor formation in host flies and subsequent metastasis [32–34].

A firm link between dysregulated asymmetric cell division and brain tumor formation has been established for central brain neuroblasts (Figure 5(a)). Indeed, a number of regulators of asymmetric cell division act as tumor suppressors in *Drosophila* neuroblasts. Thus, mutations in any one of the key asymmetrically segregated cell-fate determinants Prospero, Numb, or Brat result in brain tumors, even if these mutations are restricted to individual neuroblast clones [24, 29–32]. In the absence of any of these cell-fate determinants, the sensitive balance between self-renewal and differentiation is thought to be perturbed in the neuroblasts, leading directly or indirectly to the generation of self-renewing “tumor neuroblasts.” Uninterrupted divisions of these incorrectly specified “tumor neuroblasts” as well as failure to respond to signals that normally act in the termination of neuroblast proliferation at the end of the larval stage, result in indefinite proliferation [32–34]. Interestingly, the type II lineages which contain transit amplifying INPs appear to be especially vulnerable to tumor formation. Mutations in Brat, Numb and Earmuff in these lineages lead to a drastic and uncontrolled expansion in the number of proliferating “tumor neuroblasts.” An important feature of the brain tumors induced by mutation of asymmetric cell division regulators in neuroblasts is that their uncontrolled overgrowth potential is maintained following transplantation of mutant brain tissue into normal hosts (Figure 5(b)). Indeed, upon transplantation into wild-type adult hosts, *prospero, numb,* and *brat* mutant brain tissue form malignant tumors and metastases, and these tumors can be maintained through subsequent re-transplantation into hosts [32, 33, 94]. In this respect it is interesting to point out that human homologs of Brat [95], Numb [96], and Prospero [97] have been shown to have connections to cancer formation, and thus results obtained with studies concerning *Drosophila *tumorigenesis can be relevant for understanding mammalian tumorigenesis as well.

Tumorigenic overgrowth due to mutation in tumor suppressor genes also takes place in the optic lobes. For example, mutation of the tumor suppressor *l(3)mbt* has recently been shown to result in optic lobe overgrowth [98–100]. However, in contrast to the situation in the central brain, the primary cause of this overgrowth is not due to dysregulated proliferation of the neuroblast, it is also not a result of the asymmetric segregation of cell-fate determinants in optic lobe neuroblasts of *l(3)mbt* mutants. Rather an overproliferation of the symmetrically dividing neuroepithelial cells during their expansion phase occurs in these mutants which in turn results in the generation of an uncontrolled number of optic lobe neuroblasts. At the molecular level, this unregulated overproliferation in the optic lobes of *l(3)mbt* mutants is caused, at least in part by derepression of the target genes of the SWH signaling pathway. Accordingly, experimental repression of SWH signaling or an increased expression of its downstream targets reproduces the massive proliferation of optic lobes similar to the *l(3)mbt* mutants [100]. While extensive studies point towards the importance of SWH pathway and its downstream targets in tumorigenesis of *l(3)mbt* mutants, the tumorigenic process is likely to involve the combined imbalance of several other signaling pathways like the Notch pathway [83, 85, 101–103], the JAK-STAT pathway [38], and other developmental control pathways, some of which operate in the germline [99]. Combined together with the studies of overproliferation in central brain neuroblasts, these studies clearly show that very different cellular and molecular events can lead to the formation of neural stem cell-derived brain tumors in *Drosophila*. Thus, a different cascade of initiating events in larval brain neuroblasts and optic lobe neuroblasts finally leads to a similar outcome of overproliferating cells resulting in brain tumor formation [104, 105].

## 5. Genome-Wide Screens for Neural Stem Cell Control Elements

Given the fundamental roles of the regulators of neural stem cell differentiation and maintenance that have been shown to operate in neuroblasts during normal brain development and during abnormal brain tumor formation, an in-depth analysis of their molecular mode of action and of their molecular interaction partners is of central importance. Several successful attempts have been made in the recent past, to identify novel candidate molecules involved in neural stem cell maintenance and differentiation at the genome-wide level using both microarray techniques and transcriptional target identification [31, 106, 107]. However, the functional relevance of most of these novel candidate molecules is still unknown. A useful approach to understanding the functional relevance of such identified candidate genes, is the targeted RNAi methods used to knock down the expression of their respective genes in neural stem cells, *in vivo*, where the immediate environment and the interactions with the surrounding niche are intact. This approach is eminently feasible in *Drosophila*, since genome-wide transgenic RNAi libraries are now available which allow for candidate gene functional analysis in a tissue-specific manner [108].

In a recent genome-wide study of self-renewal in *Drosophila* neuroblasts, transgenic RNAi targeted by the binary Gal4-UAS system was used to investigate the role of all known *Drosophila* genes in neuroblasts [30, 109]. In *Drosophila,* the GAL4-UAS system [110, 111] is routinely used to analyze the function of newly found developmental genes. The technique is based on the interaction of two different kinds of transgenic strains, activator and effector lines. In an activator line the gene for the yeast transcriptional activator GAL4 is placed under the control of a specific promoter, while in the effector line the gene of interest is fused to the DNA-binding motif of GAL4 (Upstream Activating Sequences, UAS). The effector gene becomes transcriptionally active only when the flies carrying it are crossed to those of an activator line, and thereby the effector gene is directed by the pattern of expression of GAL4 in the activator. This, of course, permits the controlled ectopic expression of the effector gene. In the study by Neumüller et al., out of a total of over 12,000 analyzed genes, around 600 candidate genes, showed RNAi-dependent defects in neuroblast self-renewal or in differentiation of their neural progeny. Based on precise quantification of the resulting loss-of-function phenotype and the hierarchical clustering as well as molecular interaction data, a set of functional networks representing the molecular elements involved in the control of neuroblast self-renewal and differentiation was established. Analysis of these networks reveals key roles of interacting sets of transcriptional regulators and chromatin remodeling complexes for the control of asymmetric cell division, cytokinesis, cell growth, and differentiation in the *Drosophila* brain. It is noteworthy that the dataset obtained from this RNAi screen is highly enriched for genes expressed in mammalian stem cells and thus is likely to provide valuable genetic links for analyzing mammalian stem cells and tumor biology [109].

## 6. Conclusions

A great deal of progress has been made in understanding the cellular and molecular mechanisms that underlie proliferation and cell-fate decision in the *Drosophila* brain neuroblast model. A surprising aspect of this progress is the recent demonstration that key molecular control elements involved in asymmetric cell-fate determination in normal neuroblast lineages are also central elements in neuroblast-derived brain tumor formation. Further research in *Drosophila* and in other model systems is required to determine how the process of self-renewal and differentiation operates in normal neural stem cells and neural stem cell-derived cancer. Fortunately, the remarkable conservation of major transcriptional control and signaling pathways between flies and humans makes these studies of neural stem cells in *Drosophila* highly valuable for human stem cell biology. Thus, investigations in mammalian systems focused on the roles of those key factors for neural stem cell proliferation that have been identified in *Drosophila* are likely to be a gateway for a better understanding of many human cancers and further for developing therapeutic designs. Moreover, a sound understanding of the mechanisms underlying tumorigenic perturbations of neural stem cells is clearly a prerequisite for any potential development of neural stem cell-based therapy in humans.
